# Structural and functional brain alterations in laryngeal dystonia: A coordinate‐based activation likelihood estimation meta‐analysis

**DOI:** 10.1002/hbm.70000

**Published:** 2024-09-21

**Authors:** Nyah Kshatriya, Giovanni Battistella, Kristina Simonyan

**Affiliations:** ^1^ Department of Otolaryngology‐Head and Neck Surgery Massachusetts Eye and Ear and Harvard Medical School Boston Massachusetts USA; ^2^ Program in Speech Hearing Bioscience and Technology Harvard University Boston Massachusetts USA; ^3^ Department of Neurology Massachusetts General Hospital Boston Massachusetts USA

**Keywords:** EEG, laryngeal dystonia, MEG, meta‐analysis, MRI, neuroimaging, PET

## Abstract

Laryngeal dystonia (LD) is an isolated, task‐specific, focal dystonia characterized by intermittent spasms of laryngeal muscles impairing speech production. Although recent studies have demonstrated neural alterations in LD, the consistency of findings across studies is not well‐established, limiting their translational applicability. We conducted a systematic literature search to identify studies reporting stereotactic coordinates of peak structural and functional abnormalities in LD patients compared to healthy controls, followed by a coordinate‐based activation likelihood estimation meta‐analysis. A total of 21 functional and structural neuroimaging studies, including 31 experiments in 521 LD patients and 448 healthy controls, met the study inclusion criteria. The multimodal meta‐analysis of these studies identified abnormalities in the bilateral primary motor cortices, the left inferior parietal lobule and striatum, the right insula, and the supplementary motor area in LD patients compared to healthy controls. The meta‐analytical findings reinforce the current view of dystonia as a neural network disorder and consolidate evidence for future investigations probing these targets with new therapies.

## Graphical abstract

A coordinate‐based activation likelihood estimation meta‐analysis of 21 functional and structural neuroimaging studies in laryngeal dystonia, including 31 experiments in 521 patients and 448 healthy controls, identified abnormalities in the bilateral primary motor cortex, the left inferior parietal lobule and striatum, the right insula, and the supplementary motor area. These findings reinforce the current view of dystonia as a neural network disorder and consolidate evidence for future investigations probing these targets with new therapies.

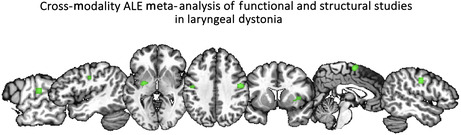

## 1 INTRODUCTION

Laryngeal dystonia (LD) is an isolated focal dystonia characterized by involuntary spasms of laryngeal muscles, causing uncontrolled voice breaks and strained, strangled, or breathy quality of voice. LD is a task‐specific disorder that predominantly affects speaking but not other types of vocalizations or laryngeal behaviors (Guiry et al., 2019). A chronically impaired ability to communicate in daily and professional settings considerably impacts various aspects of a patient's life, often leading to psychiatric comorbidities, suicidal behaviors, and lower socioeconomic status (Faham et al., 2021; Worthley & Simonyan, 2021).

Recent efforts to understand the underlying pathophysiology of LD have revealed altered brain organization in these patients compared to healthy individuals. Specifically, a series of neuroimaging studies have demonstrated abnormalities in brain function and microstructure, ranging from focal changes in selected brain regions to disorganization of the whole‐brain, large‐scale network (Battistella et al., 2016; Hanekamp & Simonyan, 2020; Haslinger et al., 2005; Mantel et al., 2020; Simonyan & Ludlow, 2010; Simonyan & Ludlow, 2012). Historically considered a basal ganglia disorder, these neuroimaging studies have helped develop the current view of dystonia as a neural network disorder, involving not only the basal ganglia but also sensorimotor cortical areas, thalamus, and cerebellum as key pathophysiological contributors (Lungu et al., 2020; Simonyan et al., 2021).

On the other hand, despite their impact on shaping the current understanding of dystonia pathophysiology, the differences in employed neuroimaging modalities, scanning protocols, analytical paradigms, and patient selection criteria between the studies have introduced discrepancies and ambiguities to the interpretation of their findings (Ramdhani & Simonyan, 2013). This, in turn, hindered a comprehensive characterization of the LD neuroimaging signature, especially for translational applications, such as probing candidate brain targets with novel therapies.

To consolidate the findings of reported neuroimaging studies and identify a consistent and reproducible set of abnormal brain regions contributing to LD pathophysiology, we conducted a systematic activation likelihood estimation (ALE) meta‐analysis of published to date functional and structural neuroimaging literature in patients with LD. The ALE methodology uses a random effects algorithm to find agreement across subject cohorts and reported activation clusters, incorporates variable uncertainty based on the cohort size, and limits the effect of a single experiment (Eickhoff et al., 2012). Thus, the ALE meta‐analytical approach allowed us to model the activation clusters as a spatial probability distribution function and map the likelihood of above‐chance convergence in the location of reported effects in LD patients.

## 2 METHODS

### 2.1 Literature search and article selection

A PubMed literature search to identify neuroimaging studies in LD patients was performed between November 14, 2022, and January 24, 2024, using Covidence systematic review software (Veritas Health Innovation, Melbourne, Australia; available at www.covidence.org) in accordance with the Preferred Reporting Items for Systematic Reviews and Meta‐Analyses (PRISMA) guidelines (Page et al., 2021). The literature search was performed using the following query: “((laryngeal AND dystonia) OR (spasmodic AND dysphonia) OR (spastic AND dysphonia)) AND ((functional AND MRI) OR (fMRI) OR (magnetic AND resonance AND imaging) OR (MRI) OR (speech AND production AND MRI) OR (resting AND state) OR (positron AND emission AND tomography) OR (PET) OR (brain AND activity) OR (brain AND activation) OR (VBM) OR (voxel‐based AND morphometry) OR (functional AND connectivity) OR (cortical AND thickness) OR (EEG) OR (electroencephalography) OR (MEG) OR (magnetoencephalography) OR (TMS) OR (transcranial AND magnetic AND stimulation)) NOT (Review[Publication Type]) Filters: Humans.”

The resultant articles were independently screened by two researchers (NK and GB) first for their title and abstract to determine their relevance for this meta‐analysis and then for the full text to extract data. The inclusion criteria were (1) an original, peer‐reviewed article, (2) no review or case studies, and (3) reported coordinates of peak abnormality in the standard coordinate system. The inter‐screener agreement rate was 0.88 (*κ* = 0.68) for the title and abstract selection and 0.90 (*κ* = 0.81) for the full‐text review. The cases of disagreement between the two screeners on the title and abstract selection (11.3% of all articles) and full‐text review (2.0% of all articles) were resolved with independent input from the senior investigator (KS).

The full‐text review of selected articles was conducted to extract the following data: (1) the study design; (2) cohort size; (3) subject demographics (age, sex, native language, handedness, and LD clinical phenotype); (4) scanner type (manufacturer, model, and strength); (5) task/condition of interest (e.g., vowel production, speech production, reading, resting, silent fixation); (6) statistical threshold of reported findings; (7) peak *xyz* coordinates of clusters derived from group comparisons; (8) cluster size, (9) standard coordinate system (Talairach–Tournoux [TT], Montreal Neurological Institute [MNI]), and (10) analytical software (e.g., AFNI, FSL, SPM, FreeSurfer, NUTMEG). In articles reporting multiple group comparisons (e.g., different LD clinical phenotypes vs. healthy controls) or more than one imaging modality, each comparison was treated as a separate experiment in the meta‐analyses. Each experiment was further categorized as a task‐production fMRI/MEG/PET/EEG, resting‐state fMRI, or structural MRI study based on the imaging modality used.

### 2.2 ALE meta‐analysis

To determine both the modality‐specific overlapping cross‐modality abnormalities in LD, four separate coordinate‐based ALE meta‐analyses were conducted as follows: (1) task‐production experiments, (2) resting‐state experiments, (3) structural experiments, and (4) all combined functional and structural experiments.

ALE meta‐analyses were performed using GingerALE software (version 3.0.2) (Eickhoff et al., 2009, 2012); statistical analysis and visualization of the resultant spatial probabilistic maps were conducted using AFNI software. First, the peak coordinates in the MNI space were converted to the AFNI standard Talairach–Tournoux space using the icbm2tal transform (Lancaster et al., 2007) and inputted into the ALE algorithm. Each coordinate was modeled as a Gaussian spatial probability distribution function with a full‐width half maximum (FWHM) derived from the number of subjects in each meta‐analysis, accounting for the spatial uncertainty of individual coordinates. Modeled activation (MA) maps were calculated by finding the union across the Gaussian functions for all coordinates in each experiment. The ALE scores were quantified as the union of MA maps across all experiments and transformed into *Z*‐scores. Statistical significance of the resultant *Z*‐scores was set at a family‐wise error (FWE)‐corrected *p* ≤ .05 with voxelwise *p* ≤ .001 and a minimum cluster size of 240 mm^3^.

## 3 RESULTS

**FIGURE 1:**
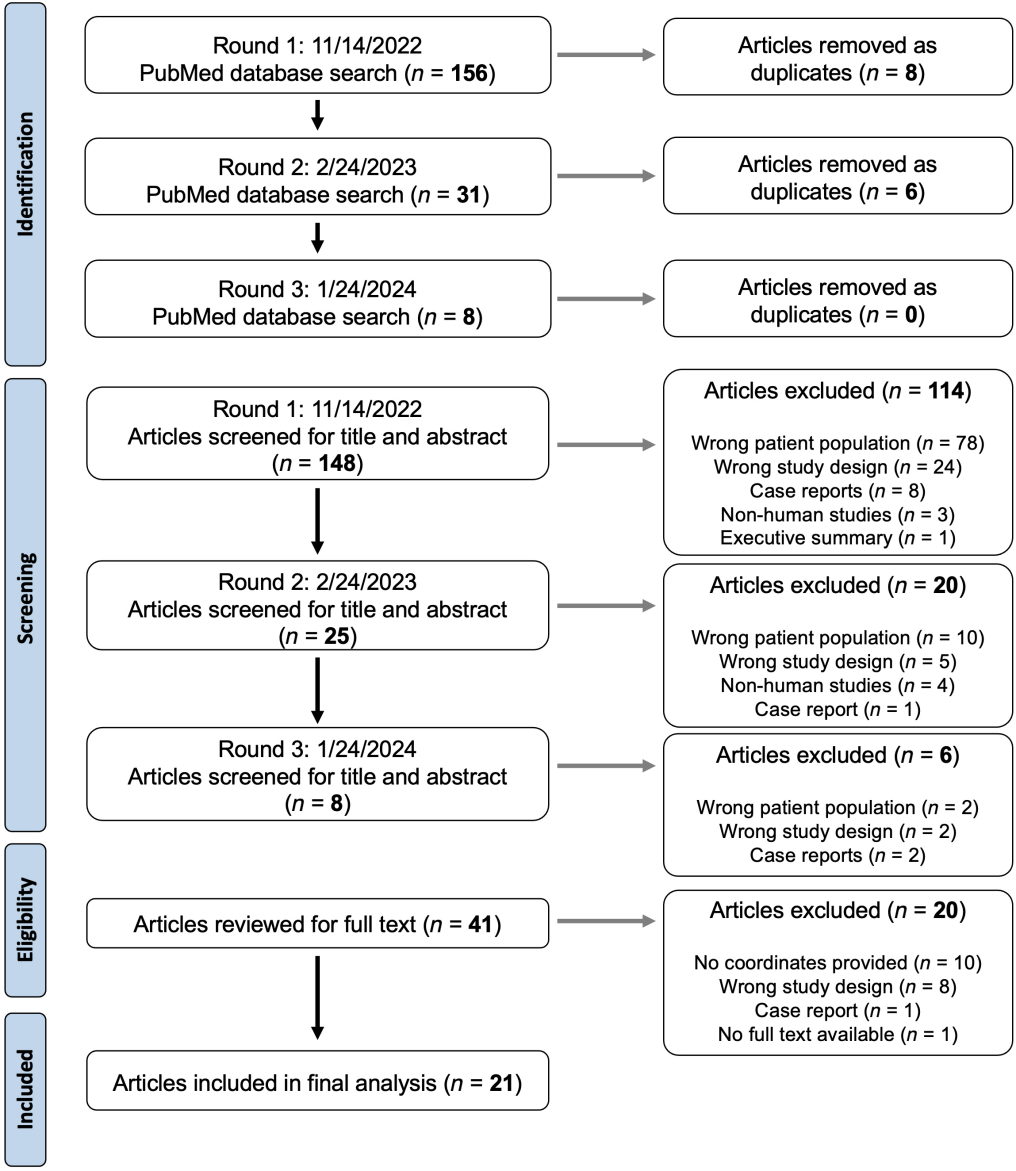
The PRISMA flowchart of study screening and selection for studies involving patients with laryngeal dystonia.

The PubMed search yielded a total of 195 articles, 14 of which were identified as duplicates and removed (Figure 1). Among the 181 remaining articles, 140 were excluded as irrelevant to this study after the title and abstract screening because of the wrong patient population (*n* = 90), wrong study design (*n* = 31), case report (*n* = 11), non‐human study (*n* = 7), and executive summary (*n* = 1). The full‐text review was conducted for the remaining 41 articles, after which 20 articles were excluded because of the lack of reported peak coordinates of brain alteration in the full‐text or supplementary material (*n* = 10), wrong study design (*n* = 8), case report (*n* = 1), and the absence of the full‐text version (*n* = 1). The remaining 21 articles reporting 31 experiments (17 task‐production fMRI/MEG/PET/EEG, 7 resting‐state fMRI, and 7 structural MRI) in a total of 521 LD patients and 448 healthy controls across all studies were included in the final meta‐analysis (see Table 1 for details).

**TABLE 1:** Characteristics of 21 studies included in the coordinate‐based meta‐analysis of abnormal brain regions in laryngeal dystonia.

Article	Participants	LD phenotype	Age	Sex	Imaging modality	Type of analysis	Task (if applicable)	Contrast (if applicable)
de Lima Xavier and Simonyan (2019)	28 LD patients with risk factors 25 LD patients without risk factors 28 HC	ADLD, ABLD	53.0 ± 11.3 54.0 ± 14.7 49.0 ± 9.7	20F/8M 17F/8M 19F/9M	fMRI	BOLD fMRI	Sentence production	Resting condition
Haslinger et al. (2005)	12 LD patients 12 HC	ADLD	52.5 ± 9 52.7 ± 7.8	5F/7M 9F/3M	fMRI	BOLD fMRI	Vowel production (/i/)	Resting condition
Kanazawa et al. (2020)	11 LD patients 11 HC	ADLD	36.7 30.9	9F/2M 9F/2M	fMRI	BOLD fMRI	Voice perception (/a/, /i/)	White noise and band noise (centered around 1000 Hz)
Khosravani et al. (2021)	21 LD patients 21 unaffected relatives 32 HC	ADLD, ABLD	56.2 ± 15.8 48.5 ± 16.0 50.2 ± 11.0	19F/2M 17F/4M 20F/12M	fMRI	BOLD fMRI	Symptom‐evoking sentence production	Resting condition
Kirke et al. (2017)	20 LD patients 20 LD/DTv patients 20 HC	ADLD, ABLD, ADLD/DTv, ABLD/DTv	54.4 ± 8.3 60.0 ± 10.1 53.8 ± 9.9	16F/4M 18F/2M 16F/4M	fMRI	BOLD fMRI	Symptom‐evoking sentence production	Resting condition
Kiyuna et al. (2014)	6 LD patients 6 HC	ADLD	24.3 30.8	5F/1M 5F/1M	fMRI	BOLD fMRI	Vowel production (/i/)	Resting condition
Kiyuna et al. (2017)	12 LD patients 16 HC	ADLD	34.3 33.1	12F/0M 16F/0M	fMRI	BOLD fMRI	Reading five‐digit numbers	Reading with no vocalization
O'Flynn and Simonyan (2022)	57 LD patients 50 HC	ADLD, ABLD, ADLD/DTv, ABLD/DTv	54.7 ± 13.4 51.0 ± 10.0	42F/15M 32F/18M	fMRI	BOLD fMRI	Symptom‐evoking sentence production	Resting condition
Simonyan and Ludlow (2010)	11 ADLD patients 11 ABLD patients 11 HC	ADLD, ABLD	50.6 ± 10.9 56.5 ± 8.7 55.7 ± 9.2	8F/3M 5F/6M 4F/7M	fMRI	BOLD fMRI	Symptom‐evoking syllable production (/i‐i/, /ihi/)	Resting condition
Simonyan and Ludlow (2012)	15 LD patients 15 HC	ADLD, ABLD	54.1 ± 10.1 49.5 ± 13.3	8F/7M 8F/7M	fMRI	BOLD fMRI	Symptom‐evoking syllable production (/i‐i/, /ihi/)	Resting condition
Termsarasab et al. (2016)	23 LD patients	ADLD, ABLD	62.7 ± 5.9	17F/6M	fMRI	BOLD fMRI	Symptom‐evoking sentence production	Resting condition
Ali et al. (2006)	9 LD patients 10 HC	ADLD	46 ± 14 39 ± 8	6F/3M 6F/4M	PET	Regional cerebral blood flow	Narrative speech production	Resting condition
Kothare et al. (2022)	15 LD patients 11 HC	ADLD, ADLD/DTv	Not reported Not reported	Not reported Not reported	MEG	Beta activity around voice onset, high‐gamma activity around voice onset	Vowel production (/a/)	
Ehrlich et al. (2023)	24 LD patients 22 HC	ADLD, ABLD, Mixed LD, VT	57.0 ± 12.5 62.2 ± 8.2	13F/11M 14F/8M	EEG	Spectral topography	Symptom‐evoking sentence production	
Battistella et al. (2016)	32 LD patients 30 HC	ADLD, ABLD	Not reported 49.7 ± 9.5	Not reported 18F/12M	Resting‐state fMRI	Resting‐state functional connectivity		
Battistella and Simonyan (2019)	20 LD patients 20 LD/DTv patients 35 HC	LD, LD/DTv (AD/AB phenotype not reported)	52.2 ± 7.3 55.5 ± 11.2 50.4 ± 10.6	13F/7M 17F/3M 22F/13M	Resting‐state fMRI	Resting‐state functional connectivity		
Bianchi et al. (2019)	8 LD, 8 FHD 16 HC	ADLD, SLD	45.3 ± 10.8 43.9 ± 11.9	8F/8M 7F/9M	Resting‐state fMRI	Resting‐state functional connectivity		
Kiyuna et al. (2017)	12 LD patients 16 HC	ADLD	34.3 33.1	12F/0M 16F/0M	Resting‐state fMRI	Resting‐state functional connectivity		
Putzel et al. (2018)	57 LD patients 30 HC	ADLD, ABLD	55.9 ± 12 49.7 ± 9.5	46F/11M 18F/12M	Resting‐state fMRI	Resting‐state functional connectivity		
Mantel et al. (2020)	14 LD patients 14 HC	ADLD	48.0 ± 14.9 Not reported	7F/7M Not reported	Resting‐state fMRI	Resting‐state functional connectivity, regional homogeneity		
Bianchi et al. (2019)	8 LD, 8 FHD 16 HC	ADLD, SLD	45.3 ± 10.8 43.9 ± 11.9	8F/8M 7F/9M	Structural MRI	Gray matter volume		
Khosravani et al. (2021)	21 LD patients 21 unaffected relatives 32 HC	ADLD, ABLD	56.2 ± 15.8 48.5 ± 16.0 50.2 ± 11.0	19F/2M 17F/4M 20F/12M	Structural MRI	Gray matter volume		
Kirke et al. (2017)	20 LD patients 20 LD/DTv patients 20 HC	ADLD, ABLD, ADLD/DTv, ABLD/DTv	54.4 ± 8.3 60.0 ± 10.1 53.8 ± 9.9	16F/4M 18F/2M 16F/4M	Structural MRI	Gray matter volume		
Ramdhani et al. (2014)	12 LD 12 WC 24 HC	Not reported	54.75 52.75 52.13	8F/4M 6F/5M 12F/12M	Structural MRI	Gray matter volume		
Simonyan and Ludlow (2012)	40 LD patients 40 HC	ADLD, ABLD	56.9 ± 10.6 52.5 ± 10.5	25F/15M 17F/23M	Structural MRI	Gray matter volume, cortical thickness		
Kostic et al. (2016)	13 LD patients 30 HC	ADLD	57.8 ± 14 58.1 ± 11	7F/6M 15F/15M	Structural MRI	Cortical surface area		

Abbreviations: ABLD, abductor laryngeal dystonia; ADLD, adductor laryngeal dystonia; BOLD, blood‐oxygen‐level dependent; DTv, dystonic tremor of voice; EEG, electroencephalography; FHD, focal hand dystonia; fMRI, functional magnetic resonance imaging; HC, healthy controls; LD, laryngeal dystonia; MEG, magnetoencephalography; MFHD, musician's focal hand dystonia; SLD, singer's laryngeal dystonia; VT, vocal tremor; WC, writer's cramp.

Among these articles, the most common imaging modality was MRI used in 27 experiments (Battistella et al., 2016; Battistella & Simonyan, 2019; Bianchi et al., 2019; de Lima Xavier & Simonyan, 2019; Haslinger et al., 2005; Kanazawa et al., 2020; Khosravani et al., 2021; Kirke et al., 2017; Kiyuna et al., 2014, 2017; Kostic et al., 2016; Mantel et al., 2020; O'Flynn & Simonyan, 2022; Putzel et al., 2018; Ramdhani et al., 2014; Simonyan & Ludlow, 2010, 2012; Termsarasab et al., 2016), followed by MEG used in 2 experiments (Kothare et al., 2022), EEG in 1 experiment (Ehrlich et al., 2023), and PET in 1 experiment (Ali et al., 2006).

Fourteen out of 21 articles reported the native language of the participants, with 12 articles using native English speakers (Battistella et al., 2016; Battistella & Simonyan, 2019; Bianchi et al., 2019; de Lima Xavier & Simonyan, 2019; Ehrlich et al., 2023; Khosravani et al., 2021; Kirke et al., 2017; O'Flynn & Simonyan, 2022; Putzel et al., 2018; Simonyan & Ludlow, 2010, 2012; Termsarasab et al., 2016), one article using native Japanese speakers (Kanazawa et al., 2020), and one article using native Serbian speakers (Kostic et al., 2016). In all but one article (Kothare et al., 2022), the experiments were conducted in right‐handed participants.

Nineteen out of 21 articles reported that participants had no other laryngeal disorders (Ali et al., 2006; Battistella et al., 2016; Bianchi et al., 2019; de Lima Xavier & Simonyan, 2019; Ehrlich et al., 2023; Haslinger et al., 2005; Kanazawa et al., 2020; Khosravani et al., 2021; Kirke et al., 2017; Kiyuna et al., 2014, 2017; Kostic et al., 2016; Mantel et al., 2020; O'Flynn & Simonyan, 2022; Putzel et al., 2018; Ramdhani et al., 2014; Simonyan & Ludlow, 2010, 2012; Termsarasab et al., 2016), and of these, 14 articles used nasolaryngoscopy to confirm the diagnosis of LD and/or the absence of other laryngeal problems. Except for two articles (Kiyuna et al., 2014, 2017), 19 articles reported that patients were recruited into the study at least 3 months after their last botulinum toxin injection and were fully symptomatic at the time of the study participation.

Nineteen out of 21 articles included patients with adductor type of LD (Ali et al., 2006; Battistella et al., 2016; Bianchi et al., 2019; de Lima Xavier & Simonyan, 2019; Ehrlich et al., 2023; Haslinger et al., 2005; Kanazawa et al., 2020; Khosravani et al., 2021; Kirke et al., 2017; Kiyuna et al., 2017; Kiyuna et al., 2014; Kostic et al., 2016; Kothare et al., 2022; Mantel et al., 2020; O'Flynn & Simonyan, 2022; Putzel et al., 2018; Simonyan & Ludlow, 2010; Simonyan & Ludlow, 2012; Termsarasab et al., 2016), 10 articles included patients with abductor type of LD (Battistella et al., 2016; de Lima Xavier & Simonyan, 2019; Ehrlich et al., 2023; Khosravani et al., 2021; Kirke et al., 2017; O'Flynn & Simonyan, 2022; Putzel et al., 2018; Simonyan & Ludlow, 2010, 2012; Termsarasab et al., 2016), 5 articles included LD patients with dystonic voice tremor (Giovanni Battistella & Simonyan, 2019; Ehrlich et al., 2023; Kirke et al., 2017; Kothare et al., 2022; O'Flynn & Simonyan, 2022), and 2 articles did not specify the LD clinical phenotype (Giovanni Battistella & Simonyan, 2019; Ramdhani et al., 2014). Only 1 article (Simonyan & Ludlow, 2010) conducted separate comparisons of adductor and abductor LD patients vs. healthy controls, and none stratified LD patients with and without voice tremor for comparisons with healthy controls.

Three out of 21 articles (Khosravani et al., 2021; Putzel et al., 2018; Simonyan & Ludlow, 2012) stated some overlap in study participants with other included articles (Battistella et al., 2016; Simonyan & Ludlow, 2010; Termsarasab et al., 2016). Importantly, two of these articles (Khosravani et al., 2021; Simonyan & Ludlow, 2012) employed different methodologies for neuroimaging data collection (structural vs. functional MRI) and analysis (voxel‐based morphometry, cortical distance estimates, and BOLD estimates during task‐production vs. resting‐state functional connectivity analysis and correlation analysis) compared to the original articles. Thus, while there is a reported partial overlap in study participants, the resultant data extracted for the meta‐analysis did not overlap. The third article (Putzel et al., 2018) stated an overlap in study participants because it used the regions of abnormal activity from another study (Battistella et al., 2016) to perform functional connectivity analysis and investigate the association between abnormal connectivity and polygenic risk of dystonia. We conducted an influence analysis (Viechtbauer & Cheung, 2010) to determine the impact of this article (Putzel et al., 2018) on the final results by removing its data from the meta‐analysis. The influence analysis found that the identified meta‐analytical regions were not derived as a result of the overlap in LD patients between these two studies. Therefore, the final meta‐analysis included all 21 articles.

### 3.1 Modality‐specific ale meta‐analysis of functional and structural studies

**FIGURE 2:**
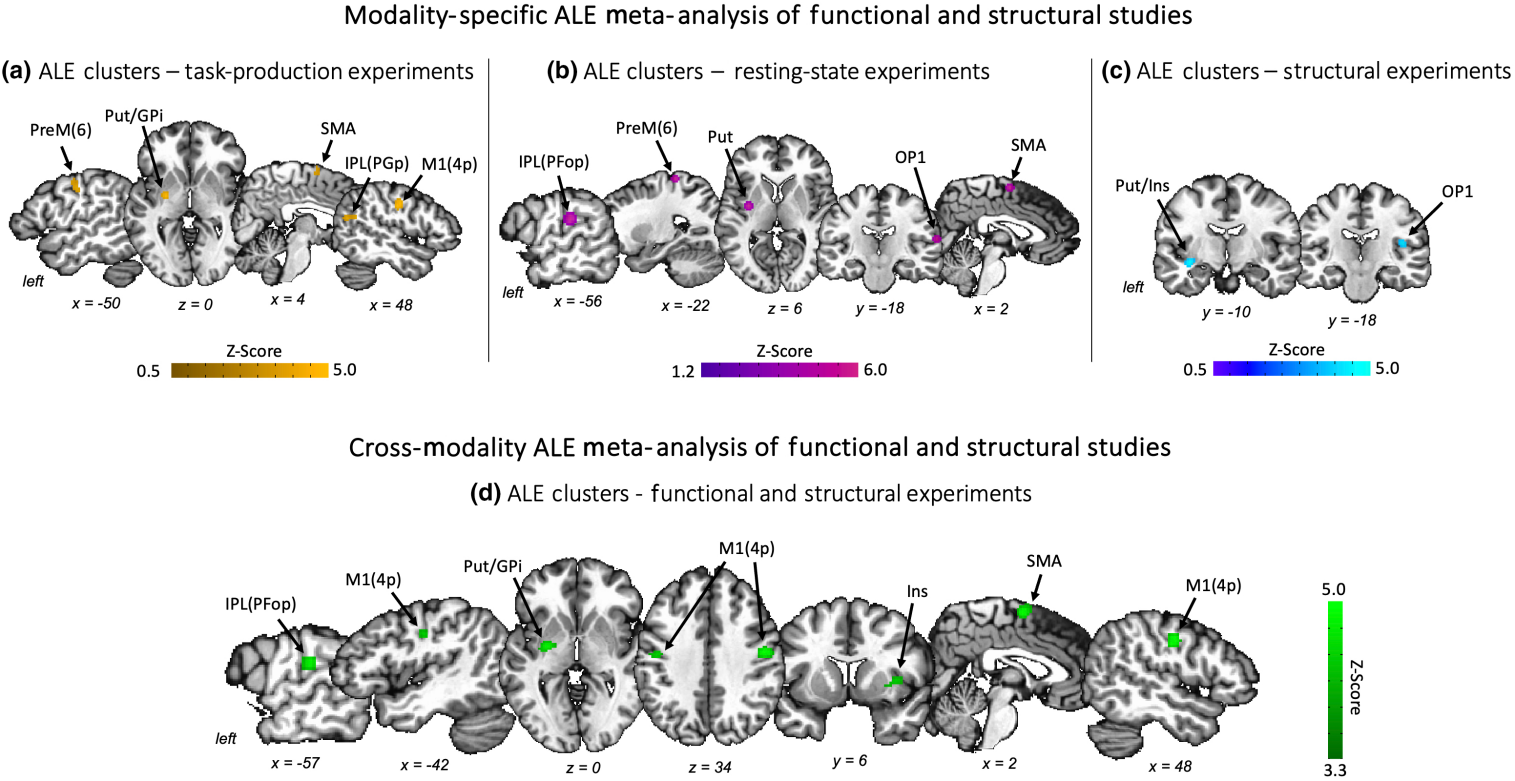
Statistically significant clusters identified using coordinate‐based ALE meta‐analysis of (a) task‐production functional activity (*yellow*), (b) resting‐state functional connectivity (*purple*), (c) voxel‐based morphometry and cortical thickness (*blue*), and (d) all functional and structural neuroimaging studies (*green*) in patients with laryngeal dystonia compared to healthy controls. Gpi, globus pallidus internus; IPL, inferior parietal lobule; Ins, insula; M1, primary motor cortex; PreM, premotor cortex; Put, putamen; SMA, supplementary motor area; STG, superior temporal gyrus.

A total of 17 experiments in 14 articles (Ali et al., 2006; de Lima Xavier & Simonyan, 2019; Ehrlich et al., 2023; Haslinger et al., 2005; Kanazawa et al., 2020; Khosravani et al., 2021; Kirke et al., 2017; Kiyuna et al., 2014, 2017; Kothare et al., 2022; O'Flynn & Simonyan, 2022; Simonyan & Ludlow, 2010, 2012; Termsarasab et al., 2016) examined task‐production brain activity in LD patients compared to healthy controls, including symptom‐evoking sentence production (*n* = 7) (de Lima Xavier & Simonyan, 2019; Ehrlich et al., 2023; Khosravani et al., 2021; Kirke et al., 2017; O'Flynn & Simonyan, 2022; Termsarasab et al., 2016), symptom‐evoking syllable production (*n* = 3) (Simonyan & Ludlow, 2010, 2012), continuous vowel production (*n* = 4) (Haslinger et al., 2005; Kiyuna et al., 2014; Kothare et al., 2022), narrative speech production (*n* = 1) (Ali et al., 2006), reading digits (*n* = 1) (Kiyuna et al., 2017), and voice perception (*n* = 1) (Kanazawa et al., 2020). Among these, 13 experiments used BOLD fMRI (de Lima Xavier & Simonyan, 2019; Haslinger et al., 2005; Kanazawa et al., 2020; Khosravani et al., 2021; Kirke et al., 2017; Kiyuna et al., 2014, 2017; O'Flynn & Simonyan, 2022; Simonyan & Ludlow, 2010, 2012; Termsarasab et al., 2016), two experiments used MEG to examine the oscillatory activity in the beta and high‐gamma frequency bands (Kothare et al., 2022), one experiment used H_2_^15^O PET with regional cerebral blood flow (rCBF) (Ali et al., 2006), and one experiment used EEG to examine spectral topography (Ehrlich et al., 2023). A total of 194 coordinates were extracted from these experiments and used in the ALE meta‐analysis. Five significant clusters were located in the right primary motor cortex (area 4p) and IPL (area PGp), the left premotor cortex (area 6) and putamen extending to GPi, and the bilateral SMA at FWE‐corrected *p* ≤ .05 (Figure 2a, Table 2).

**TABLE 2:** Probabilistic clusters of functional and structural brain abnormalities in LD patients.

Brain region	Cluster peak coordinates, *x y z*	Cluster size (mm^3^)	Cluster peak Z‐value
Task‐production activation			
R primary motor cortex (area 4p)	46, −10, 32	888	5.41
L putamen, extending to globus pallidus internus	−24, −8, 0	352	4.69
R/L supplementary motor area	4, −2, 60	336	3.69
R inferior parietal lobule (area PGp)	48, −60, 20	280	3.89
L premotor cortex (area 6)	−50, −10, 40	264	3.75
Resting‐state functional connectivity			
L inferior parietal lobule (area PFop)	−60, −26, 22	872	6.81
L putamen	−30, −6, 6	632	5.53
R parietal operculum (area OP1)	62, −18, 14	480	5.51
R/L supplementary motor area	2, −4, 56	480	5.51
L premotor cortex (area 6)	−22, −32, 62	480	5.51
Structural			
L putamen, extending to insula	−32, −10, −6	320	4.47
R parietal operculum (area OP1)	42, −18, 14	272	4.19
All modalities			
L putamen, extending to globus pallidus internus	−30, −6, 6	1640	5.23
R primary motor cortex (area 4p)	48, −10, 34	1112	5.52
L inferior parietal lobule (area PFop)	−60, −26, 22	952	6.05
R/L supplementary motor area	2, −4, 58	864	5.50
R insula	36, 4, 6	472	4.09
L primary motor cortex (area 4p)	−42, −12, 34	296	4.15

Abbreviations: L, left; R, right.

Additionally, 7 experiments in 6 articles (Battistella et al., 2016; Giovanni Battistella & Simonyan, 2019; Bianchi et al., 2019; Kiyuna et al., 2017; Mantel et al., 2020; Putzel et al., 2018) investigated functional connectivity using resting‐state fMRI in LD patients compared to healthy controls. Five experiments computed functional connectivity using independent component analysis (ICA) to isolate resting‐state networks implicated in LD pathophysiology, including the sensorimotor network (SMN), frontoparietal network (FPN), auditory network (AN), and central executive network (CEN) (Battistella et al., 2016; Giovanni Battistella & Simonyan, 2019; Bianchi et al., 2019; Mantel et al., 2020; Putzel et al., 2018). One experiment (Kiyuna et al., 2017) conducted a seed‐to‐voxel functional connectivity analysis with seeds derived from regions previously demonstrated to have abnormal activity in LD patients. Another experiment (Mantel et al., 2020) computed regional homogeneity, characterizing the local temporal coherence between each voxel and its nearest neighbor. The ALE meta‐analysis of 31 coordinates derived from these studies found 5 significant clusters located in the left IPL (area PFop), premotor cortex (area 6), and putamen, the bilateral SMA, and the right parietal operculum (area OP1) at FWE‐corrected *p* ≤ .05 (Figure 2b, Table 2).

Finally, 7 experiments in 6 articles (Bianchi et al., 2019; Khosravani et al., 2021; Kirke et al., 2017; Kostic et al., 2016; Ramdhani et al., 2014; Simonyan & Ludlow, 2012) reported structural differences between LD patients and healthy controls. Among these, 5 experiments examined gray matter volume (Bianchi et al., 2019; Khosravani et al., 2021; Kirke et al., 2017; Ramdhani et al., 2014; Simonyan & Ludlow, 2012), one experiment investigated cortical thickness (Simonyan & Ludlow, 2012), and one experiment computed cortical surface area (Kostic et al., 2016). The ALE meta‐analysis of 53 coordinates from these structural MRI experiments found two significant clusters, located in the left putamen, extending to the insula and the right parietal operculum (area OP1) at FWE‐corrected *p* ≤ .05 (Figure 2c, Table 2).

### 3.2 Cross‐modality ALE meta‐analysis of functional and structural studies

Combining all 31 functional and structural experiments in 21 articles, a total of 278 coordinates were used to identify common functional and structural alterations across all imaging modalities in LD patients. The ALE meta‐analysis found six clusters located in the bilateral primary motor cortex (area 4p), the left inferior parietal lobule (IPL, area PFop) and putamen extending to the globus pallidus internal segment (GPi), and the right insula and supplementary motor area (SMA) at FWE‐corrected *p* ≤ .05 (Figure 2d, Table 2).

## 4 DISCUSSION

We performed a systematic meta‐analytical investigation of published to date literature reporting functional and structural neural alterations in LD patients to define the most commonly affected brain regions that likely contribute to the pathophysiology of this disorder. Our key findings point to the presence of distributed abnormalities involving not only the striatum and primary motor cortex but also associative cortical regions, such as the IPL, premotor cortex, SMA, parietal operculum, and insula. These meta‐analytical findings align well with the prevailing notion of dystonia as a neural network disorder (Lungu et al., 2020; Simonyan et al., 2021) and consolidate evidence for future investigations probing these targets as biomarkers for LD differential diagnostics and new therapies.

The role of both the primary motor cortex and basal ganglia has been well‐established in dystonia pathophysiology and is now supported by our meta‐analytical findings. Identified bilateral abnormalities in the primary motor cortex correspond to the location of the laryngeal motor cortex (LMC) (Bouchard et al., 2013; Simonyan, 2014; Simonyan & Horwitz, 2011). The LMC is an essential hub of motor execution within the speech production network, with wide‐ranging connections to other cortical and subcortical regions that are hierarchically involved in sensory processing and feedback, sensorimotor integration, and motor planning during speaking (Fuertinger et al., 2015; Simonyan & Fuertinger, 2015; Valeriani & Simonyan, 2021). Through direct connections to brainstem laryngeal motoneurons, the LMC regulates the final cortical motor output during speech production (Simonyan & Horwitz, 2011). Its abnormal activity likely directly impacts the dystonic pattern of laryngeal muscle activation observed during speech production in LD patients.

As a prominent subcortical structure in both the speech production network and dystonia pathophysiology, the striatum plays a critical role in the initiation of intended actions and the suppression of unwanted, competing motor patterns. An imbalance between the facilitatory and inhibitory effects of the direct and indirect basal ganglia pathways may manifest as an overall decrease of inhibition throughout the basal ganglia–thalamo–cortical circuitry (Hallett, 2011; Simonyan et al., 2017) and subsequently contribute to altered motor cortical execution in dystonia patients. In the current meta‐analysis, functional and structural abnormalities were consistently found within the somatotopic representation of the larynx in the striatum (Simonyan & Jurgens, 2003) across all examined modalities, hence, reinforcing the notion that specific changes in this region remain a prevalent pathophysiological feature of dystonia.

However, despite their apparent involvement in dystonia pathophysiology, the previous attempts to alleviate dystonic symptoms using invasive or non‐invasive neuromodulation of the basal ganglia and motor cortex have yielded highly heterogeneous results. For example, while deep brain stimulation (DBS) of basal ganglia targets has been effective in treating a variety of movement disorders, including generalized, segmental, and cervical dystonia (Jacksch et al., 2022; Lee et al., 2019; Yin et al., 2022), its benefits in LD patients remain variable, ranging from no changes to worsening of symptoms (Isaias et al., 2009). Efforts to apply non‐invasive brain stimulation over the primary motor cortex in patients with focal dystonia have similarly resulted in variable outcomes, largely dependent on patient cohorts, stimulation parameters, and targeted coordinates (Morrison‐Ham et al., 2022). The inconsistent speech outcomes of stimulation targeting the motor cortex and basal ganglia suggest that these regions, although integral to LD pathophysiology, may not be of a primary importance for therapeutic interventions in these patients. Conversely, the associative regions identified through this meta‐analysis might represent alternative targets for neuromodulation.

To that end, our meta‐analyses highlighted abnormalities in premotor, parietal, and insular regions in LD patients, all of which have been demonstrated to have strong functional and structural connections with the LMC (Kumar et al., 2016; Simonyan et al., 2009) and associated with extrinsic risk factors for this disorder (de Lima Xavier & Simonyan, 2019). The involvement of an array of these cortical regions in the dystonic network of LD is also consistent with the task‐specificity of the disorder, characterized by disruptions of learned, finely skilled movements (Ramdhani et al., 2014).

Specifically, premotor regions are highly involved in motor preparation of voluntary vocal commands, with voice‐related neural activity in the SMA found to precede activity in the motor cortex (Galgano & Froud, 2008). The SMA has also been attributed to higher‐level functions related to speech production, including initiation and timing control, inhibitory control, complex sequencing, and task switching, and represents a key component in cortical networks as well as in the basal ganglia–thalamo–cortical loop (Hertrich et al., 2016). Disruptions of premotor cortical functional activity and connectivity in LD may imply disrupted motor preparation, planning, initiation, and sequencing mechanisms. With direct connections to primary motor regions, these abnormalities likely contribute significantly to the manifestation of LD symptomatology.

The current meta‐analytical findings also strengthen evidence for the substantial role of IPL alterations in LD pathophysiology. The IPL consists of the supramarginal and angular gyri, the former of which is thought to be important in sensorimotor integration (Guenther, 2016). Functional activation of the IPL has been shown during experimental conditions to evoke a mismatch between sensory expectations and feedback, including delayed auditory feedback, unexpected somatosensory feedback perturbations, and transitions in visual, auditory, and tactile stimuli, implying that this region is essential for sensory feedback control during the production of speech motor tasks (Downar et al., 2000; Golfinopoulos et al., 2011; Hashimoto & Sakai, 2003). Abnormal activation and functional connectivity of the IPL have been previously implicated in various forms of task‐specific focal dystonia, including LD, musician's dystonia, and writer's cramp (Battistella & Simonyan, 2019; Bianchi et al., 2019; Delnooz et al., 2012; Gallea et al., 2016; Maguire et al., 2020; Putzel et al., 2018), suggesting that processing of somatosensory feedback during the production of highly skilled tasks may be perturbed in these patients. This notion is supported by a phenomenology of *geste antagoniste* or sensory tricks in LD patients, in which perturbations to peripheral sensory inputs, including touching the throat or humming before speaking, are able to temporarily alleviate LD symptoms (Blitzer et al., 2018). In a recent study of effective connectivity, the IPL has been found to exhibit abnormal top‐down influences on the premotor cortex and putamen, supporting the hypothesis that altered parietal–premotor and parietal–putaminal information transfer may precede motor cortical changes within the dystonic network (Battistella & Simonyan, 2019).

Additionally, our meta‐analysis identified abnormalities in the insula, an important outflow hub involved in cognitive and sensorimotor behaviors (Hanekamp & Simonyan, 2020; Menon & Uddin, 2010). Through a combination of lesion and neuroimaging studies, the insula has been demonstrated to play an important yet diverse and ambiguous role in speech motor control (Baldo et al., 2011; Bohland & Guenther, 2006; Dronkers, 1996). Multiple insular subdivisions have been shown to exhibit non‐overlapping structural connections to cortical regions involved in articulatory modulation and motor preparation, auditory and phonological processes, and motor execution (Battistella et al., 2018). A reorganization of these subdivisions, specifically those highly connected to motor regions, and a loss of the insular hub as part of structural and functional connectome have been previously reported in LD patients (Battistella et al., 2017; Hanekamp & Simonyan, 2020). Our findings suggest that altered insular‐cortical connectivity in LD likely contributes to disrupted information flow between motor planning and sensorimotor regions, potentially playing a larger than initially assumed role in LD clinical symptomatology (Hanekamp & Simonyan, 2020).

While the current meta‐analysis consolidated the reported neuroimaging findings in LD, its limitations should be acknowledged. The meta‐analytical cohort was comprised of LD patients with different clinical subtypes of the disorder. Among these, the majority (90.5%) of articles included adductor LD, nearly half (47.6%) of articles included abductor LD and about a quarter (23.8%) of articles included LD with dystonic tremor. The demographics of the meta‐analytical study cohort follow the typical demographics of this disorder (Blitzer et al., 2018), which suggests that the reported results may be applicable to the general patient population. However, given the substantially smaller proportions of LD patients with abductor and tremor subtypes, only 1 study reporting comparisons between adductor/abductor LD and healthy controls, and none examining LD with or without dystonic tremor vs. healthy controls, separate meta‐analyses of LD cohorts stratified by these clinical phenotypes were not feasible.

In conclusion, using ALE meta‐analysis, we identified a robust set of functionally and structurally abnormal brain regions in LD that spatially converge across neuroimaging modalities, scanner types, and methodological paradigms. Our findings confirm the presence of extensive network‐wide disruptions underlying this disorder. In addition to the basal ganglia and primary motor cortical alterations, other cortical areas, including premotor, parietal, and insular regions, are likely to represent the major pathophysiological nodes of the LD neural network. The next series of studies is warranted to discern the directionality of abnormal influences within this pathophysiological network, as well as the relationships between functional and structural changes, which would help clarify the pathophysiological mechanisms that trigger the development of LD. Future studies may also probe the identified meta‐analytical brain region as biomarkers for differential diagnosis of LD from other neurological disorders or non‐neurological conditions mimicking dystonic voice. Furthermore, these regions might represent new targets for novel therapeutic interventions using centrally acting medications or neuromodulation for restoration of neural network function.

## CONFLICT OF INTEREST STATEMENT

None declared.

## Data Availability

Data are available upon request. Upon the acceptance of this manuscript, the research data used in this study will be archived in the figshare public repository. Analytic codes used in this study will be publicly available at https://simonyanlab.meei.harvard.edu/resources.
